# *Notes from the Field:* Environmental Investigation of a Multistate Salmonellosis Outbreak Linked to Live Backyard Poultry from a Mail-Order Hatchery — Michigan, 2018

**DOI:** 10.15585/mmwr.mm675152a5

**Published:** 2019-01-04

**Authors:** Margaret C. Hardy, Scott A. Robertson, Jennifer Sidge, Kimberly Signs, Mary Grace Stobierski, Kelly Jones, Marty Soehnlen, Lisa Stefanovsky, Adeline Hambley, Joshua M. Brandenburg, Haley Martin, A.C. Lauer, Patricia Fields, Lia Koski, Lauren M. Stevenson, Kristy L. Pabilonia, Megin C. Nichols, Colin A. Basler, Efrain M. Ribot, Kelley B. Hise

**Affiliations:** ^1^Laboratory Leadership Service, CDC; ^2^Division of Foodborne, Waterborne and Environmental Diseases, National Center for Emerging and Zoonotic Infectious Diseases, CDC; ^3^Epidemic Intelligence Service, CDC; ^4^Division of Foodborne, Waterborne and Environmental Diseases, National Center for Emerging and Zoonotic Infectious Diseases, CDC; ^5^Bureau of Epidemiology and Population Health, Michigan Department of Health and Human Services; ^6^Bureau of Laboratories, Michigan Department of Health and Human Services; ^7^Ottawa County Department of Public Health, Holland, Michigan; ^8^Veterinary Diagnostic Laboratories, Colorado State University, Fort Collins, Colorado.

In the United States, contact with live poultry has been linked to 70 *Salmonella* outbreaks resulting in 4,794 clinical cases since 2000 (*1*). Environmental sampling to confirm the outbreak strain at poultry hatcheries that supply backyard flocks is conducted infrequently during investigations; therefore, the source of the outbreak is rarely identified. On June 12, 2018, the Michigan Department of Health and Human Services requested assistance from CDC to investigate risk factors for *Salmonella* infection linked to live backyard poultry originating at a mail-order hatchery in Michigan (hatchery A). This hatchery supplies young poultry (poults) to backyard flocks through direct sale to flock owners and via feed stores. At the start of the investigation, traceback had linked 24 clinical cases of *Salmonella enterica* serotype Enteritidis to exposure to live poultry from hatchery A. Whole genome sequencing analysis of the clinical isolates revealed that they were closely related (within 0–15 alleles) by whole genome multilocus sequence typing to environmental isolates sampled from shipping containers originating from hatchery A at retail outlets in several states.

Environmental sampling for *Salmonella* was conducted at hatchery A on June 19. Collectors were briefed on priorities and techniques on the day of sampling to ensure consistency. The four sampled areas were prioritized to ensure that the majority of samples were collected from the following areas: 1) hatching environment (liners inside egg hatchers and incubators and inside and outside surfaces of egg hatchers and incubators); 2) preshipping area (swabbing of work surfaces); 3) resident breeding stock environment (laying boxes, bedding, and food or water containers); and 4) trucks used for live poultry and egg transportation onsite and offsite. Shoe covers worn by the sampling team inside hatchery buildings also were tested to sample the environment.

Using best practices for biosecurity (*2*), two sample collectors and two data recorders conducted environmental sampling. Published procedures for environmental collection were reviewed (*3*), and hatchery A was sampled using three swab types: sterile polyurethane culture swabs in liquid Amies agar gel, sterile wooden swabs, and sterile gauze squares. Samples were collected from chick box liners and bedding and placed in sterile whirl pack bags and sterile collection cups, respectively. Samples were transported and delivered at ambient temperature to Michigan Department of Health and Human Services within 6 hours. Samples were cultured and characterized through polymerase chain reaction, followed by pulsed-field gel electrophoresis and whole genome sequencing of isolates.

Among 45 samples collected, *Salmonella* was identified in four (9%) (Table). Three isolates collected from the same building were identified as *Salmonella enterica* serotype Typhimurium, and one isolate from poults in the preshipping area was closely related to the outbreak strain (differing by 1–3 alleles by whole genome multilocus sequence typing. Epidemiologic and laboratory investigations are ongoing.

**TABLE:** Results of environmental sampling for *Salmonella* by priority sample area — hatchery A, Michigan, June 2018

Priority sample area*^,†^	No. of samples	Culture results		
		*S.* Typhimurium,^§^ no. (%)	*S*. Enteritidis,^¶^ no. (%)	Negative, no. (%)
Hatching environment	11	0 (—)	0 (—)	11 (100)
Preshipping area	20	0 (—)	1 (5)	19 (95)
Breeding stock	12	3 (25)	0 (—)	9 (75)
Trucks	2	0 (—)	0 (—)	2 (100)
**Total**	**45**	**3 (7)**	**1 (2)**	**41 (91)**

* Hatching environment = liners inside egg hatchers and incubators, and inside and outside surfaces of egg hatchers and incubators; preshipping area = swabs of work surfaces; resident breeding stock environment = laying boxes, bedding, and food or water containers; trucks = vehicles used for live poultry and egg transportation onsite and offsite.

^†^ Shoe covers worn by the sampling team inside hatchery buildings were also tested to sample the environment.

^§^ Positive samples collected in the same building from chick box liners, shoe covers worn inside the building, and bedding where chicks were housed.

^¶^ Collected from male Cornish Rock poults in the preshipping area; this isolate was found to be closely related to the outbreak strain by whole genome sequencing analysis.

The investigation confirmed the presence of the outbreak strain at hatchery A. Environmental sampling at poultry hatcheries should be considered as part of an outbreak response. This investigation supported the use of identified priority areas for systematic sampling for *Salmonella* at poultry hatcheries.
